# Characterisation of ATP-Dependent Mur Ligases Involved in the Biogenesis of Cell Wall Peptidoglycan in *Mycobacterium tuberculosis*

**DOI:** 10.1371/journal.pone.0060143

**Published:** 2013-03-21

**Authors:** Tulika Munshi, Antima Gupta, Dimitrios Evangelopoulos, Juan David Guzman, Simon Gibbons, Nicholas H. Keep, Sanjib Bhakta

**Affiliations:** 1 Department of Biological Sciences, Institute of Structural and Molecular Biology, Birkbeck, University of London, London, United Kingdom; 2 Department of Pharmaceutical and Biological Chemistry, UCL School of Pharmacy, London, United Kingdom; French National Centre for Scientific Research – Université de Toulouse, France

## Abstract

ATP-dependent Mur ligases (Mur synthetases) play essential roles in the biosynthesis of cell wall peptidoglycan (PG) as they catalyze the ligation of key amino acid residues to the stem peptide at the expense of ATP hydrolysis, thus representing potential targets for antibacterial drug discovery. In this study we characterized the division/cell wall (*dcw*) operon and identified a promoter driving the co-transcription of *mur* synthetases along with key cell division genes such as *ftsQ* and *ftsW*. Furthermore, we have extended our previous investigations of MurE to MurC, MurD and MurF synthetases from *Mycobacterium tuberculosis*. Functional analyses of the pure recombinant enzymes revealed that the presence of divalent cations is an absolute requirement for their activities. We also observed that higher concentrations of ATP and UDP-sugar substrates were inhibitory for the activities of all Mur synthetases suggesting stringent control of the cytoplasmic steps of the peptidoglycan biosynthetic pathway. In line with the previous findings on the regulation of mycobacterial MurD and corynebacterial MurC synthetases via phosphorylation, we found that all of the Mur synthetases interacted with the Ser/Thr protein kinases, PknA and PknB. In addition, we critically analyzed the interaction network of all of the Mur synthetases with proteins involved in cell division and cell wall PG biosynthesis to re-evaluate the importance of these key enzymes as novel therapeutic targets in anti-tubercular drug discovery.

## Introduction

Tuberculosis (TB) is a one of the leading causes of human mortality from infectious diseases with an estimated 1.4 million deaths globally in 2011 [1]. Control of TB has become much harder with the recent emergence of extensively-drug resistant TB (XDR-TB) strains, as there is virtually no effective drug available for their treatment [2]. Therefore new drugs with novel mechanisms of action are urgently required to tackle the spread of drug-resistant TB strains.

*Mycobacterium tuberculosis,* the causative pathogen of TB, is extremely tolerant to chemical agents and this feature is attributed to its remarkably impermeable cell wall, which consists of a covalently linked mycolyl-arabinogalactan-peptidoglycan (mAGP) complex. The cell wall peptidoglycan (PG), which is unique to bacteria, provides a rigid support that gives the cell its shape and maintains its turgidity [3]. PG biosynthesis is the target of several clinically useful antibiotics (cycloserine, bacitracin, vancomycin and β-lactams) and its regulation is thought to be correlated with critical biological processes such as bacterial cell elongation and division, thus validating the pathway as a prospective source of vulnerable targets for antibacterial drug discovery [4]. To date not a single clinically available drug has been reported to target ATP-dependent Mur ligases (Mur synthetases), which are key enzymes of the PG biosynthetic pathway. Our findings on the inhibition of MurE synthetase in *M. tuberculosis* have highlighted this group of enzymes as potential anti-mycobacterial targets [5], [6], [7].

During PG biosynthesis, the soluble muropeptide precursors are synthesized in the mycobacterial cytoplasm and are then translocated across the cytoplasmic membrane to the periplasmic space where they undergo transglycosylation and transpeptidation reactions carried out by the penicillin binding proteins (PBPs) [8], to form mature PG. Mur synthetases are key central enzymes in the cytoplasmic steps of PG biosynthesis. MurC initiates the formation of the stem peptide by adding L-alanine (L-Ala) to the carboxyl group of uridine-diphospho-*N*-acetyl-muramic acid (UDP-MurNAc), while MurD, MurE and MurF sequentially add D-glutamate (D-Glu), *meso*-diaminopimelate (*m*-DAP) and D-alanine-D-alanine (D-Ala-D-Ala) forming the stem pentapeptide UDP-MurNAc-L-Ala-γ-D-Glu-*m*-DAP-D-Ala-D-Ala [9]. The presence of *m*-DAP and D-Ala-D-Ala residues in the soluble PG precursor is essential for the later crosslinking of adjacent muropeptide residues in the final steps of PG biosynthesis [3].

Mur synthetases have been characterized in several microorganisms [10], [11], [12]; however knowledge of the structure, function and regulation of these enzymes is still fragmented in *M. tuberculosis*. Mahapatra *et al* (2000) reported that MurC was able to incorporate glycine (Gly) and L-Ala to UDP-MurNAc in both *M. tuberculosis* and *M. leprae* [13]. Our investigation of the structural and functional characterization of MurE from *M. tuberculosis* [14], [15] revealed that MurE was only active in the presence of its specific natural substrates: uridine-diphospho-*N*-acetylmuramoyl-L-alanine-D-glutamate (UDP-MurNAc-L-Ala-D-Glu), *m*-DAP and ATP. In this study, we comprehensively examine and compare the activity of all Mur synthetases in *M. tuberculosis* with respect to their natural substrates.

All of the four genes for the *M. tuberculosis* Mur synthetases are positioned close to each other in the division/cell wall (*dcw*) cluster, which also contains key cell division genes. Since cell elongation and septum formation during cell division involves recruitment of both cell wall PG biosynthetic and cell division proteins [3], their co-transcription may be important for the proliferation of mycobacteria. In this study we report the analysis of the *dcw* operon and demonstrate for the first time the promoter driving the co-transcription of *mur* synthetases and the adjacent cell division genes. Moreover, growing evidence that these groups of proteins interact to form a complex during cell division, further prompted us to investigate the network of interaction of the proteins of the *dcw* operon.

In order to understand the protein-protein interaction network of MurC, D, E, and F synthetases, we also analyzed other key protein partners which are involved in their regulation and/or PG biogenesis. These included the serine-/threonine protein kinases (STPKs), PknA and PknB that have been reported to regulate cell wall biosynthesis, cell division, pathogenicity and survival during various stress conditions through phosphorylation/dephosphorylation of their target protein substrates [16]. We also investigated proteins involved in the production of the amino acid substrates for Mur synthetases, such as glutamate racemase (MurI), diaminopimelate epimerase (DapF) and D-alanine:D-alanine ligase (DdlA) [17], [18], [19]. Furthermore, as the amino sugar units of mycobacterial muropeptides have uniquely been found to be both *N*-acetylated and *N*-glycolylated [20], it was therefore intriguing to determine at which step during the PG biosynthesis the NamH protein [21] caused this modification. Combining basic bioinformatic data analysis with our *in vivo* protein-protein interaction experimental results, we attempted to uncover an endogenous interaction network for these proteins.

## Materials and Methods

### Bacterial strains, plasmids and chemicals

*Escherichia coli* DH5α (Promega) was used for cloning, and *E. coli* BL21(DE3)/pLysS and *Pseudomonas putida* KT2442 for overexpressing *M. tuberculosis* Mur synthetases. pET28b(+), pET43.1b(+) (Novagen) and pVLT31 were used for the overexpression of mycobacterial proteins in *E. coli* and *P. putida*, respectively. *Mycobacterium smegmatis* mc^2^155 was used as host, while the pUAB100 and pUAB200 plasmids were used as the vectors for *in vivo* protein-protein interaction studies. All restriction endonucleases were purchased from New England Biolabs. All other media and chemicals were purchased from Sigma-Aldrich unless mentioned otherwise.

### Cloning of *M. tuberculosis* genes

The *murC* (Rv2152c) and *murF* (Rv2157c) genes were amplified from *M. tuberculosis* H37Rv genomic DNA using Phusion hot start DNA polymerase and primers listed in table S1, and cloned into pET28(b)+ vector at NdeI/BamHI sites to obtain pSBC2 and pSBC4 respectively. pVLT31, derived from pMMB207, does not encode for a fusion-tag [22]; hence pSBC1 [15], pSBC2 and pSBC4 were digested with XbaI/HindIII to give ∼2.0 kb fragments containing the ribosome binding site (RBS), His-tag, a thrombin cleavage site and the genes of interest, which were then sub-cloned into pVLT31 at the same sites to obtain p31E, p31C and p31F respectively. *M. tuberculosis murD* (Rv2155c) was cloned in frame with NusA using BamHI/HindIII sites in the pET43.1(b)+ vector, which also contains a His-tag, and thrombin and enterokinase cleavage sites in the linker region (Table S2), to obtain p43D. The clones were selected in *E. coli* DH5α, confirmed by sequencing and then used to transform *E. coli* BL21(DE3)/pLysS and electro-competent *P. putida* KT2442 in the presence of kanamycin (50 µg/mL) and chloramphenicol (34 µg/mL) for pSBC1, pSBC2 and pSBC4, ampicillin (100 µg/mL) and chloramphenicol (34 µg/mL) for p43D and tetracycline (12.5 µg/mL) and rifampicin (10 µg/mL) for p31C, p31E and p31F respectively.

To construct bait clones for protein interaction studies, *murC, murD, murE* (Rv2158c), *murF* and *nat* (Rv3566) were PCR amplified from *M. tuberculosis* H37Rv genomic DNA using the primers listed in table S1 and cloned into integrating vector pUAB200 at MfeI/ClaI sites, except for MurE where the MfeI site was present within the gene so it was replaced with EcoRI, to obtain murC200, murD200, murE200 murF200 and nat200. Similarly, for prey constructs, *pknA* (Rv0015c), *pknB* (Rv0014c), *murI* (Rv1338), *dapF* (Rv2726c), *ddlA* (Rv2981c) *namH* (Rv3818), Rv2160c, *ftsW* (Rv2154c), *ftsQ* (Rv2151c), *ftsZ* (Rv2150c), *sepF* (Rv2147c) and *wag31* (Rv2145c) genes were PCR amplified and ligated into the episomal vector pUAB100 (restriction enzyme sites are underlined in the primers as shown in table S1) to produce pknA100, pknB100, murI100, dapF100, ddlA100, namH100, Rv2160-100, ftsW100, ftsQ100, ftsZ100, sepF100, and wag100 respectively. The pUAB200 and pUAB100 constructs were amplified in *E. coli* DH5α and then selected in the presence of kanamycin (50 µg/mL) and hygromycin (150 µg/mL) respectively. All clones were confirmed by DNA sequencing.

### Over-expression and purification of *M. tuberculosis* Mur synthetases

One litre Luria Bertani (LB) *P. putida* cultures, supplemented with tetracycline and rifampicin, were grown at 30°C, induced with 1 mM IPTG at OD 0.8 and incubated for a further 16 h for expression of the recombinant protein. For MurD, *E. coli* cultures were supplemented with ampicillin and chloramphenicol and grown at 37°C, induced with 0.5 mM IPTG at OD 0.6 and incubated for 16 h at 18°C. The cells were harvested and lysed by sonication (10 µm amplitude, 5×30 s pulse with 1 min cooling interval) in chilled lysis buffer [25 mM Tris. HCl, 300 mM NaCl, 10% glycerol and 5 mM β-mercaptoethanol (pH 8.0)]. Expression of recombinant proteins was confirmed by western blot using alkaline phosphatase conjugated His-tag antibodies. The cytoplasmic fraction was separated by centrifugation at 50,000×g for 1 h at 4°C. For purification, the cytoplasmic fraction containing the recombinant protein was applied to a pre-equilibrated Ni^2+^-NTA column, followed by washing with lysis buffer containing 25 mM imidazole and eluted with 200 mM imidazole. The peak fractions were analyzed by 12.5% SDS-PAGE. The pure fractions were pooled, concentrated by ultrafiltration (10 kDa cut-off) at 4°C and cleaved with thrombin at a concentration of 2 units per mg of protein overnight at 4°C. The concentrated proteins were further purified by size exclusion chromatography using a Sephacryl 200 column (GE Healthcare) attached to an Akta chromatographic system (Amersham Biosciences) in 25 mM Tris-HCl, 100 mM NaCl and 1 mM β-mercaptoethanol buffer (pH 8.0). For MurD, ion-exchange chromatography was carried out using a HiTrap™ IEX XL column (GE Healthcare) to further purify the protein. The concentration of purified proteins was estimated using a Nanodrop 1000 spectrophotometer (Thermo Fisher Scientific) and proteins were stored in 10% glycerol at −80°C with minimal loss of activity.

### Functional assay for *M. tuberculosis* Mur synthetases

Enzyme activities were assayed by measuring the release of orthophosphate following enzymatic ATP hydrolysis using the Pi ColorLock Gold kit (Innova Biosciences) as reported earlier [15]. The optimum buffer and pH was determined using Tris–HCl (between pH 7.0–9.0), Bis-tris–propane–HCl (pH 7.0–9.0), HEPES-NaOH (pH 7.0–8.0) and Bis-tris-HCl (pH 7.0–8.0). The thermal stabilities of the purified enzymes were determined by measuring enzyme activities over a linear range of temperatures from 10 to 70°C. The optimized assay was performed in a final volume of 50 µL using 100–200 ng of enzyme in the presence of 50 mM Bis-tris buffer (pH 8.5), 5 mM MgCl_2_, 1 mM ATP, 1 mM of amino acids L-Ala, D-Glu or D-Ala-D-Ala and 0.1 mM of the relevant UDP-MurNAc substrates at 37°C for 30 min. The absorbance was measured at 635 nm using a FLUOstar Omega plate reader (BMG Labtech). To control for any non-enzymatic hydrolysis of ATP, the background absorbance of the reaction mixture without enzyme was measured and subtracted from the absorbance values with enzyme. Each reaction was performed in triplicate and the standard deviation of the mean was calculated and is shown as error bars. The steady-state kinetic parameters were determined for each of the three substrates, and their Michaelis constant (*K*_M_) and maximal reaction velocity (*V*_max_) were determined by non-linear regression analysis based on the Michaelis-Menten equation. The substrate specificity experiments were conducted by assessing the ATPase activity in the presence of 1.5 mM concentration of different amino acids, 1 mM concentration of different nucleotides and 0.5 mM concentration of different UDP-sugars.

### HPLC analysis

The ligase activity was evaluated on an Agilent 1100 Series HPLC instrument (Agilent Technologies, Palo Alto, CA) at a flow rate of 0.5 mL/min with absorbance continually measured at 220 and 268 nm as reported earlier [6]. The stationary phase used for the separation was an octadecylsilane (RP-18) Jones chromatography column (4.6 mm×250 mm×5 µm). Elution was carried out isocratically with a buffer of 50 mM ammonium formate at pH 4.0. A calibration curve was constructed for UDP-MurNAc (rt  = 7.5 min) and UDP-MurNAc-L-Ala (rt  = 11.6 min), resulting in linear responses at 268 nm with correlation factors (R^2^) of 0.99835 and 0.99895, respectively. The assay conditions were exactly the same as in the colorimetric assay. After incubation for 30 min, the reaction was stopped by heating for 10 minutes at 100°C. The resulting sample was centrifuged and transferred to HPLC vials with glass inserts. The amount of product formed was calculated from the values of the area under the peaks and quantified with the calibration curve. The peaks were further analysed by liquid chromatography – mass spectrometry (LC-MS) performed on a Finnigan LCQ ion trap mass spectrometer (ThermoFinnigan, San Jose, CA) coupled to an Alliance Waters 2695 HPLC Separations Module (Milford, MA, USA).

### *dcw* operon analysis

Total RNA was extracted from *Mycobacterium bovis* BCG (three biological replicates in duplicates) that were grown to an OD_600_ of 0.4–0.6 using the GTC method [23]. cDNA was synthesised using the Super Script Reverse Transcriptase III kit (Invitrogen) according to the manufacturer's instructions. Mock cDNA samples were also prepared where Super Script III Reverse Transcriptase was replaced by water and used as a negative control to detect genomic DNA (gDNA) contamination. The overlapping regions of the genes in the *dcw* cluster were amplified from the cDNA using *Taq* DNA polymerase (NEB) and primers outlined in table S3. A positive control using genomic DNA extracted from *M. bovis* BCG was also included. The PCR products were analysed in a 1.2% agarose gel following standard procedures.

To screen for the presence of a promoter driving the *dcw* operon in *M tuberculosis*, the regions upstream of the putative operon were investigated. These regions were between ORFs *Rv2159c-Rv2158c (murE)* (P1) and *Rv2162c (PE_PGRS38)-Rv2161c* (P2). The P1 and P2 regions were cloned into the BamHI site of the transcriptional-translational vector pYUB76 using primers as shown in table S1, driving expression of a downstream *lacZ* gene, and screened for blue colonies of *M. smegmatis* mc^2^155 on kanamycin (50 µg/mL) and X-gal (50 µg/mL) plates. The confirmation of promoter activity was done by measuring β-galactosidase activity in the presence of 2-nitrophenyl-β-D-galactopyranoside (ONPG) as a substrate [24].

### Protein-protein interaction assay

*M. smegmatis* was co-transformed with each pair of bait (*murC/D/E/F/nat*) and prey (*pknA, pknB, murI, dapF, ddlA, namH, Rv2160c, ftsW, ftsQ, ftsZ, sepF, wag31* as well as *murC/D/E/F*) constructs. The double transformants were selected on Middlebrook 7H11 medium (MB7H11) supplemented with 0.2% tween-80, 0.5% glycerol, 0.5% glucose, kanamycin (25 µg/mL) and hygromycin (50 µg/mL).

To perform the interaction assay, the same numbers of equal sized colonies were suspended in PBS followed by streaking of cells onto MB7H11 media containing kanamycin, hygromycin and trimethoprim (TMP) (12.5 µg/mL). Plates without TMP were the growth control. The plates were incubated at 37°C for 7 days and growth was observed. All bait and prey constructs alone had minimum bactericidal concentrations of less than 6.25 µg/mL for TMP. The pUAB200::pUAB100 (both consisting leucine-zipper GNC4) and inhA200::fabD100 co-transformant, reported earlier as interacting partners [25] were used as positive controls, while unrelated protein AccD6 (Rv2247) in combination with MurC (murC200::accD6-100), arylamine *N*-acetyltransferase/Nat (Rv3566) with cell division proteins (nat200::ftsW/ftsQ/ftsZ/sepF/wag31-100) and MurC with empty vector (murC200::pUAB100) were taken as negative controls. All interactions were repeated twice for confirmation. For quantitative analysis, two-fold serial dilutions of TMP were made in a 96-well plate using Middlebrook 7H9 medium (MB7H9) with tween-80 and glycerol, and then an equal number of co-transformants (∼10^6^ colony-forming units) were added to each well. The plates were incubated for 16 h at 37°C after which 30 µL of 0.01% (w/v) of freshly prepared resazurin was added to each well. Samples were assayed in triplicate and after 16 h of incubation at 37°C, a change in color intensity from blue to pink was observed, and fluorescence was measured at λ_exc_560/λ_emi_590 nm using a fluorimeter (FLUOstar Omega plate reader, BMG Labtech).

## Results

### Mur synthetases purified in active and appropriately folded form

**Figure 1:**
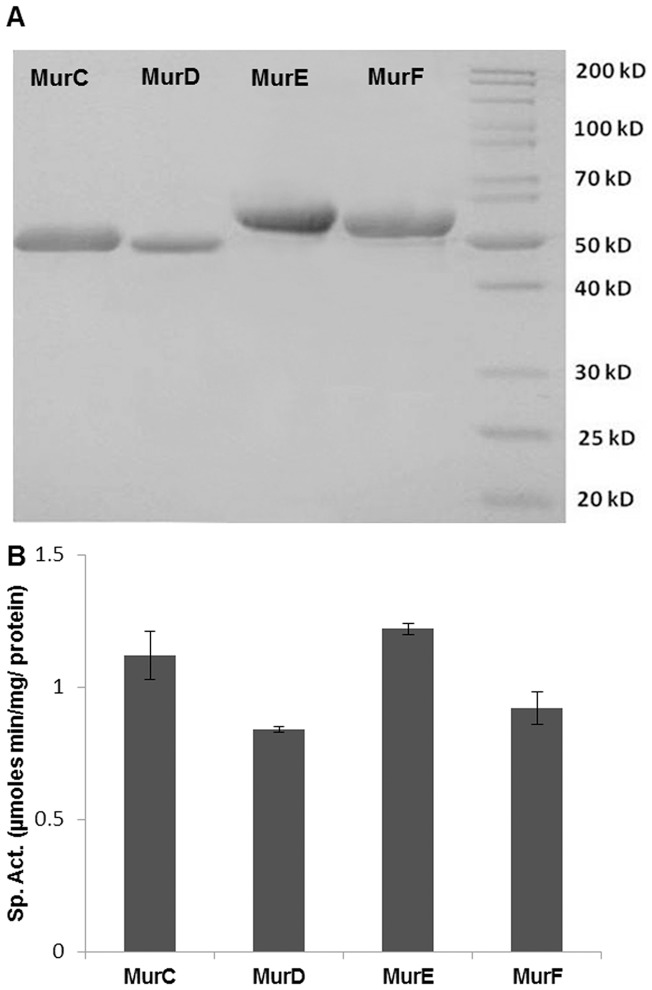
Analysis of purified recombinant *M. tuberculosis* Mur synthetases. (A) SDS-PAGE analysis of MurC (lane 1), MurD (lane 2), MurE (lane 3) and MurF (lane 4) with protein molecular weight markers (lane 5) and (B) specific activity of each protein. Error bar at 1 SD based on assays conducted in triplicate for each protein.

Achieving sufficient quantities of pure MurC and MurF proteins using expression in *E. coli* presented a challenge, as the native *E. coli* proteins, SlyD and ArnA which were identified by protein sequencing, co-eluted with the Mur synthetases. Switching to *P. putida* strain KT2442 [22], achieved elevated levels of significantly purer hexa His-tagged MurC and MurF recombinant proteins expressed at 30°C compared to *E. coli* BL21(DE3)/pLysS expressed protein at either 18°C or 30°C (data not shown). Using *P. putida* also improved the purity of the MurE protein compared to that obtained with *E. coli* previously [15]. The presence of a His-tag in the pVLT31 plasmid in frame with the proteins of interest allowed western blot analysis and purification using Ni^2+^-NTA to get homogenous enzymes as visualised by SDS-PAGE (Fig. 1A). The purity obtained for each protein using *P. putida* as a host for over-expression was considerably higher (∼95%) in comparison to that achieved with *E. coli* (∼85%). MurD, however posed a further solubility problem using the above strategies, but was successfully over-expressed and purified as a fusion protein with *E. coli* NusA, known for its ability to confer solubility to insoluble proteins [26]. MurC (51.5 KDa), MurD (51.0 KDa), MurE (55.3 KDa) and MurF (51.6 KDa) (table S2) were purified to achieve yields of 12 mg, 4 mg, 12 mg and 8 mg per liter of culture respectively. They ran as monomeric proteins on gel filtration chromatography. The colorimetric activity assay [15] and CD analysis (data not shown) confirmed that all Mur synthetases were active and correctly folded. The specific activities of MurC, MurD, MurE and MurF were estimated to be 1.2, 0.8, 1.3 and 0.9 µmoles of inorganic phosphate formed by enzyme catalysis per min per mg of protein (Fig. 1B).

### Temperature and pH affect enzyme activity and stability

The ATPase assay for Mur synthases was conducted in a 96-well half-area plate in 50 μL reaction volumes as reported earlier for MurE [15]. After testing several concentrations of the proteins (10–500 ng), the assay was carried out with a final concentration of 100 ng of MurC and MurF, and 150 ng of MurD. The stability of all proteins was notably affected by temperature and ionic strength. The effect of temperature was studied between 10 and 70°C and stable activity was observed below 40°C. When the proteins were incubated at higher temperature, the activity of all three proteins, especially MurC diminished across a temperature range of 45 to 70°C (Fig. S1 [Ai]). The optimum temperatures for activity were between 35 and 40°C for all four proteins. Moreover, almost 50% loss of activity was observed for the proteins within a week when stored at room temperature (25°C), with MurC showing a much steeper decrease in activity than MurD and MurF (Fig. S1 [Aii]). Optimum activity was achieved using Tris-HCl buffer at pH 8.0 for MurC and MurF, and Bis-tris propane at pH 8.5 for MurD, however, as the difference was not large, Bis-tris propane was used for assaying all three proteins (Fig. S1 [B]). The activity of the proteins in HEPES and Bis-tris buffers was considerably low at these pHs (<50%).

### Substrate specificity and kinetic parameters of MurC, D and F

**Figure 2:**
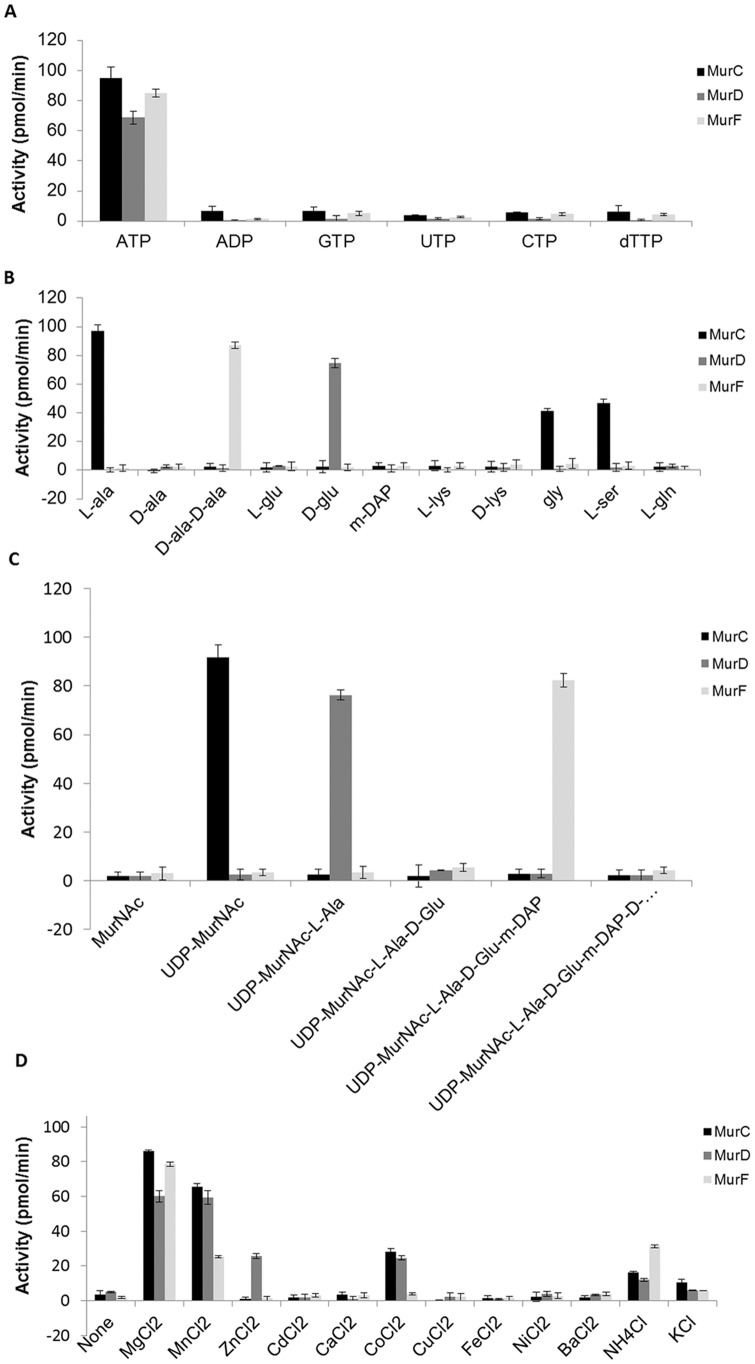
Determination of substrate specificities of Mur synthetases. Different (A) Nucleotides (B) Amino acids (C) Uridine sugars and (D) divalent and monovalent cations (at 5 mM concentration) were tested to analyze their specificities for MurC, MurD and MurF synthetases. X-axis represents different substrates used. Y-axis, in all the cases, represents the amount of P_i_ released in pmol/min.

We carried out characterisation of MurC, D and F to complement our previously published data on MurE [14], [15]. The specificities of MurC, MurD and MurF for their respective substrates were investigated. Among the nucleotides and UDP sugars tested, only ATP and UDP-MurNAc (for MurC)/UDP-MurNAc-L-Ala (for MurD)/UDP-MurNAc-L-Ala-γ-D-Glu-m-DAP (for MurF) exhibited activity (Fig. 2A and 2C). Furthermore, MurD and MurF showed activity only with D-Glu and D-Ala-D-Ala respectively among the amino acids examined. However, in the case of MurC, the activity was observed with L-Ala as well as Gly and L-Ser (Fig. 2B). This result was confirmed by HPLC which showed the presence of new peak different to that of the L-Ala-ligated product at a retention time (rt) of 6.8 min for L-Ser, whereas the peak for the Gly-ligated product co-eluted with the UDP-MurNAc substrate at 7.3 min. Further analysis by LC-MS revealed the presence of deprotonated anions in negative-mode MS for the product peaks at the expected mass/charge ratio (*m/z*) of 749.0, for UDP-MurNAc-L-Ala, 764.9 *m/z* for UDP-MurNAc-L-Ser and 735.0 *m/z* for UDP-MurNAc-Gly (Fig. S2). The *K*_M_ values obtained for L-Ala, L-Ser and Gly were 43.0, 99.7 and 146.6 µM respectively, being higher than those reported earlier for L-Ala and Gly [13]. This difference could be attributed to the different methods used to assay the activity of MurC.

Several monovalent and divalent cations were also tested for their effect on MurC, MurD and MurF activities, as in the absence of added metal ions there was very little detectable product. The L-Ala, D-Glu, *m*-DAP and D-Ala-D-Ala adding activity of MurC, MurD, MurE [15] and MurF respectively, was found to be highly dependent on the Mg^2+^ concentrations as has also been seen with other microorganisms [27], [28]. MgCl_2_ exhibited maximum activity, followed by MnCl_2_ which in the case of MurD was comparable to Mg^2+^. Other divalent cations that could be substituted for Mg^2+^ or Mn^2+^ were Co^2+^ (for MurC and MurD) and Zn^2+^ (for MurD), although the activities were considerably lower than those observed for Mg^2+^ or Mn^2+^ (Fig. 2D). Monovalent ions K^+^ and NH_4_^+^ were slightly better than some divalent ions at replacing Mg^2+^. Monovalent ions were previously found to stimulate the activity of MurD in *E. coli* and *Haemophilus influenzae* [28], although this may have been enhancing the rate in the presence of Mg^2+^. MgCl_2_ showed the highest activity at 5 mM for all three proteins, followed by MnCl_2_ at 10 mM for MurC and 5 mM for MurD and MurF. The activity of these proteins was reduced by >80% when using an increased concentration (>20 mM) of MgCl_2_ or MnCl_2_ (data not shown).

**Figure 3:**
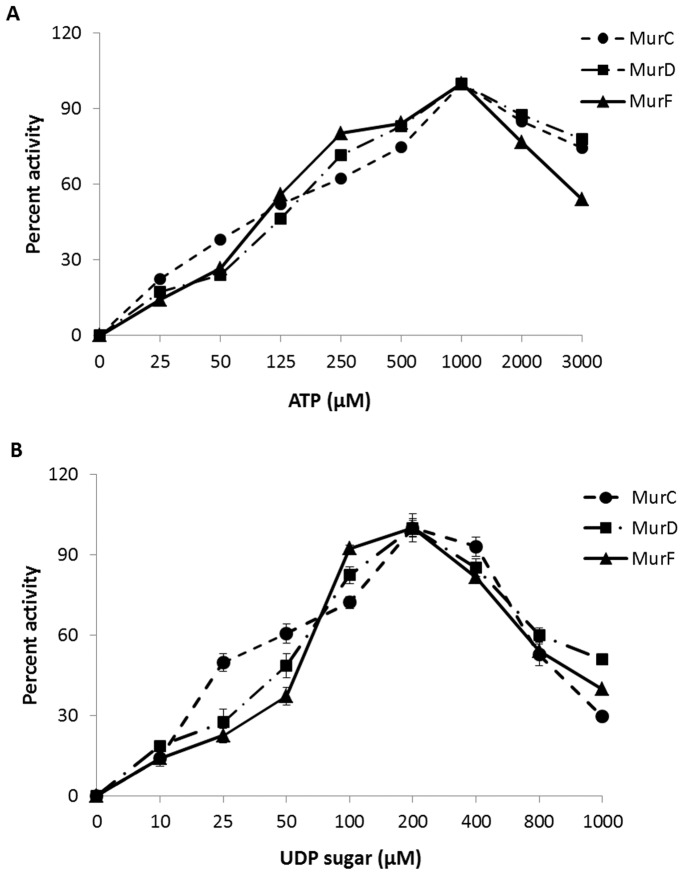
Estimation of optimal substrate concentration for Mur synthetases. Inhibition curves obtained for MurC, MurD and MurF synthetases with (A) ATP and (B) their respective uridine sugars. X-axis represents substrate concentration used and Y-axis is the percent inhibition calculated for each concentration.

Kinetic experiments showed that the optimal concentration of ATP and UDP sugar was 1000 µM and 200 µM respectively for steady state kinetics at 37°C (Fig. 3). Using twice these concentrations reduced the activity of the enzymes to around 80% of the maximal activity. This finding is in agreement with the earlier published data for *E. coli* MurD [28] and *Pseudomonas aeruginosa* MurE [29]. The Michaelis constant (*K*_M)_ values obtained for *M. tuberculosis* MurC (Table 1), were found to be lower for UDP-MurNAc and ATP and similar for L-Ala in comparison to the published values for *E. coli* MurC [30]. MurD on the other hand exhibited higher *K*_M_ values for UDP-MurNAc-L-Ala whereas ATP and D-Glu values were comparable to those obtained for *E. coli* MurD [28]. However, kinetic analysis of *M. tuberculosis* MurD by Barreteau *et al* [31] showed higher *K*_M_ values, which might be attributed to the difference in assay methods used for analysis. Similarly the *K*_M_ values for ATP and UDP-MurNAc-L-Ala-γ-D-Glu-m-DAP were obtained for MurF, but were much lower than those published for either *Staphylococcus aureus* or *E. coli* MurF [11], [32]. Furthermore, all three synthetases showed at least a 2-fold higher specificity (*k*_cat_/K_M_) towards their sugar substrates than their ATP or amino acid substrates, which is expected for bigger substrates compared to small substrates, as more interactions (electrostatic, hydrogen bonds, van der Waals) are possible throughout the length of the substrate.

**Table 1:** Kinetic parameters of MurC, MurD and MurF proteins for endogenous substrates.

	MurC	MurD	MurE¤	MurF
*K*_M_^ATP^ (µM)	67.7±1.1	106.4±0.1	120	92.3±0.2
*K*_M_^UNAM/UMA/UMAG/UMT^ * (µM)	23.5±0.5	53.3±0.1	40	64.5±0.2
*K*_M_^L-Ala/D-Glu/mDAP/D-Ala-D-Ala^ (µM)	43.0±0.9	85.1±0.2	69	78.1±0.2
*V*_max_ (µmol min^−1^mg^−1^)	1.2±0.1	0.8±0.1	1.2	0.9±0.01
*k*_cat_ (sec^−1^)	1.0±0.01	0.8±0.2	1.2	1.4±0.5
*k*_cat_/*K*_M_^ATP^ (mM)	15.1±0.6	7.5±0.1	10	15.3±0.1
*k*_cat_/*K*_M_^UNAM/UMA/UMAG/UMT^ (sec^−1^ mM^−1^)	44.8±0.3	15.1±0.1	30	21.7±0.2
*k*_cat_/*K*_M_^L-Ala/D-Glu/mDAP/D-Ala-D-Ala^ (sec^−1^ mM^−1^)	23.9±0.23	9.4±0.12	17.4	18.1±0.3
*k*_cat_ (*E. coli*) (sec^−1^)	15.4#	15.5§	-	13.1¥
*K*_M_^L-Ser^ (µM)	99.7±0.5			
*K*_M_^Gly^ (µM)	146.5±1.6			
*k*_cat_^ L-Ser^ (sec^−1^)	1.2±0.01			
*k*_cat_^ Gly^ (sec^−1^)	1.53±0.1			
*k*_cat_/*K*_M_^ L-Ser^ (sec^−1^ mM^−1^)	12.5±0.02			
*k*_cat_/*K*_M_^ Gly^ (sec^−1^ mM^−1^)	10.47±0.1			

* UNAM: UDP-MurNAc; UMA: UDP-MurNAc-L-Ala; UMAG: UDP-MurNAc-L-Ala-D-Glu; UMT: UDP-MurNAc-L-Ala-γ-D-Glu-m-DAP.

¤ [15].

# [27].

§ [67].

¥ [32].

### Characterisation of *dcw* operon

**Figure 4:**
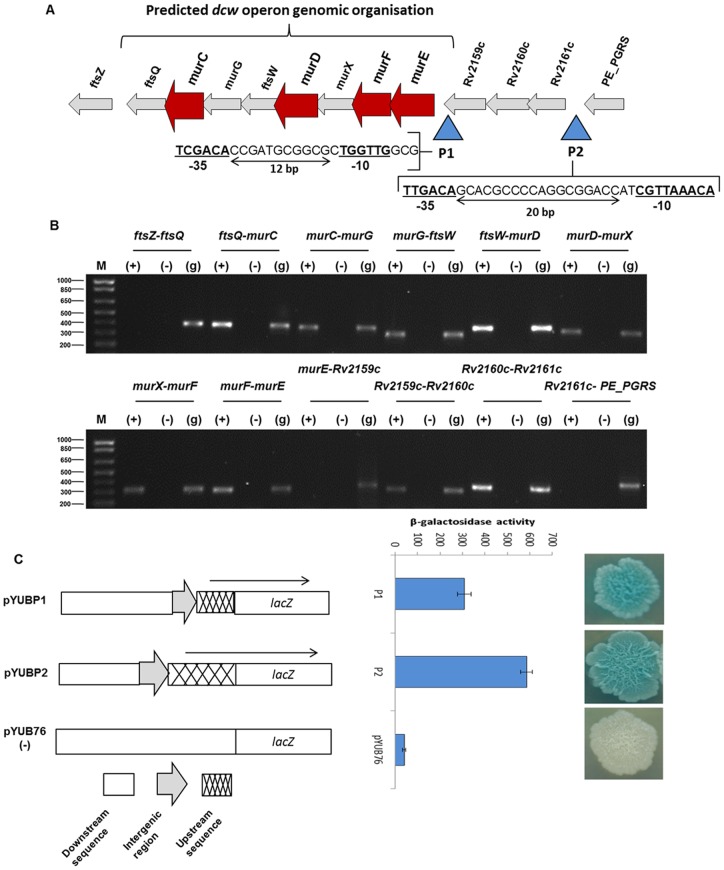
*dcw* operon analysis. (A) Representation of the *M. tuberculosis* genomic region (2408385–2424838), showing ORFs and gaps, and highlighting regions upstream of the *dcw* operon screened for the presence of a promoter driving the operon. (B) cDNA analysis for identifying boundaries of the *dcw* operon. (C) Promoter analysis by cloning into the promoter-less vector pYUB76 and β-galactosidase assay for confirmation of promoter activity.

The entire reverse direction (27.9 kb) region containing the *dcw* gene cluster in *M. tuberculosis* was studied and the overlaps or gaps were identified between each open reading frame. Total RNA was extracted and cDNA prepared from *M. bovis* BCG as the *dcw* region is identical to that in *M. tuberculosis* in the intergenic regions and shows only five single nucleotide changes in the coding sequences. To confirm the span of the *dcw* operon (Fig. 4A), reverse transcriptase PCR (RT-PCR) was carried out from *PE_PGRS38* to *ftsZ* genes using primers designed to overlap adjacent genes and amplify intergenic regions. No amplification was observed between the *PE_PGRS38-Rv2161c*, *Rv2159c-murE* and *ftsQ-ftsZ* regions (Fig. 4B). This indicated that the *Rv2161c-Rv2160c-Rv2159c* region and the cluster upstream of *murE* as far as *ftsQ* were on separate mRNA transcripts, and that *murE* was the first gene of the *dcw* operon in *M. tuberculosis*.

To screen for the promoter driving the *dcw* operon, the P1 and P2 regions upstream of the putative operon were cloned in front of *lacZ* in *M. smegmatis*. Blue colonies were observed for both regions, indicating the presence of promoters. A β-galactosidase assay was carried out for each, which further confirmed these results as shown in figure 4C. This indicated that the promoter in the P1 region drives the transcription of the *dcw* operon with *murE* as a first gene, while the promoter in the P2 region is responsible for driving the upstream region of *Rv2161c-Rv2160c-Rv2159c*, where the product of *Rv2160c* has been identified as a hypothetical protein showing homology to the putative TetR-family transcriptional regulator, which is currently under investigation. The P1 and P2 regions were screened for −10 and −35 sequences against the previously identified promoters in mycobacteria species [33], [34], and were identified as showing consensus to those recognized by a plethora of sigma factors present in *M. tuberculosis*, *M. bovis* and *M. paratuberculosis* (Fig. 4A). The promoter (P1) for the *dcw* operon was identified 123 nucleotides upstream of the GTG start site of *murE* within the *Rv2159c* gene. This promoter has TGGTTG as −10 sequences and TCGACA as −35 sequences with a 12 nucleotides gap between them. Furthermore, the promoter in the P2 region driving the *Rv2161c-Rv2160c-Rv2159c* was identified 32 nucleotides upstream of the transcription start site of *Rv2161c* with CGTTAAACA as the −10 sequences and TTGACA as the −35 sequences and with a 20 nucleotides gap between them.

### Mur synthetases interact with cell division and other regulatory proteins

**Figure 5:**
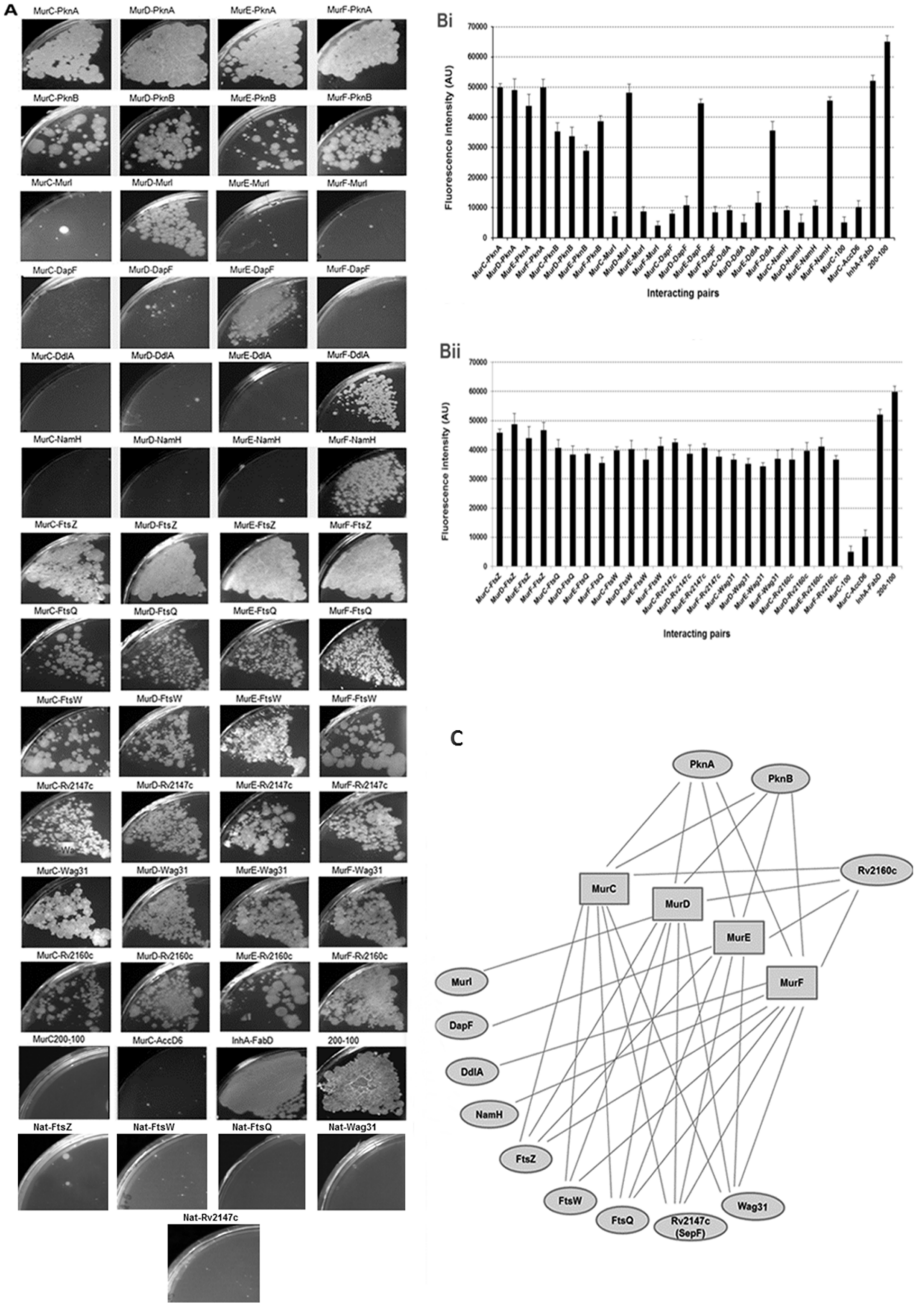
Protein-protein interaction studies of *M. tuberculosis* Mur synthetases. (A) Interaction using an M-PFC where growth on TMP plates at 12.5 µg/mL concentration indicated a positive protein-protein interaction, (B) Quantitation of M-PFC interactions by the resazurin assay and (C) representation of final interaction results. Each interaction, by both methods, was assayed in triplicate.

We screened for interacting protein partners using the mycobacterial protein fragment complementation (M-PFC) method [35], which scores for resistance to TMP owing to the regeneration of functional murine dihydrofolate reductase (mDHFR) activity from its sub-domains encoded on separate plasmids (pUAB100 and pUAB200). Significant growth was observed at a concentration of 12.5 µg/mL of TMP for interacting Mur synthetases and PknA/B proteins. The density of growth for the interaction between MurC, D, E and F with PknA was similar to the positive control, representing a strong interaction. A comparatively weaker interaction was observed between Mur synthetases and PknB (Fig. 5A). All M-PFC interaction results were further quantified by determination of the fluorescence intensity of viable cells using resazurin which gives semi-quantitative estimates of the level of interaction (Fig. 5B).

Interesting results were observed when screening Mur synthetases for interacting partners amongst themselves as well as the neighbouring cell division proteins in the genome. As shown in figure 5, the cell division proteins FtsZ, FtsW, FtsQ, Wag31 and Rv2147c (ortholog of SepF), were all found to interact with the Mur synthetases. To confirm the specificity of these interactions, the cell division proteins were also screened against the *M. tuberculosis* Nat protein. No interaction was observed between Nat and any of the cell division proteins. Furthermore, the absence of interaction among the Mur synthetases acted as an additional negative control supporting the validity of the M-PFC system for interactions of these proteins. Proteins such as MurI, DapF, DdlA and the product of *Rv3818* (*namH* homologue), which are responsible for providing substrates during PG biosynthesis, only interacted with specific Mur synthetases (Fig. 5). Whilst MurI (converts L-glutamate to D-glutamate) and DapF (transforms LL-diaminopimelate to *meso*-diaminopimelate) were found to interact with MurD and MurE, respectively, DdlA (ligates D-Ala and D-Ala) and the NamH homologue (which converts the *N*-acetyl form of UDP-sugar to the *N*-glycolyl form) interacted only with MurF (Fig. 5). In addition the product of *Rv2160c*, which is present on the operon immediately upstream of the *dcw* operon, also showed interaction with all of the Mur synthetases.

## Discussion

Structural and functional differences have been determined between the cell wall PG of mycobacteria and other bacteria [36], [20], [37], nevertheless, the biosynthesis of *M. tuberculosis* PG is generally assumed to be similar to that of *E. coli* [38]. The cell wall PG biosynthetic enzymes MurC, D, E and F in *E. coli* and *M. tuberculosis* share limited sequence identity but have several highly conserved regions that map primarily to the active site residues of the enzymes.

Although we were able to produce sufficient amounts of MurE [15] and MurC when over-expressed using the pET28b+ vector in the *E. coli* system, attempts to over-express and purify MurD and MurF using the same strategy were unsuccessful. However, using *P. putida* as a host considerably improved both the quantity and purity of MurF as well as MurC and MurE, preventing contamination by the histidine-rich co-eluting *E. coli* proteins SlyD (20 histidine residues) and ArnA (25 histidine residues). This could be due to the fact that the homologues of these proteins have less histidine residues in *P. putida*, and therefore they bind less strongly to nickel resins, thus helping to overcome the limitations of using *E. coli* as a standard heterologous system. The *P. putida* system has also been successfully used earlier to purify the *M. tuberculosis* HsaD protein [39], suggesting that the codon usage in the *P. putida* strain is more favorable for overexpressing certain *M. tuberculosis* proteins. MurD however, remained insoluble using the above strategy. Furthermore, the co-expression of MurD with *M. tuberculosis* trigger factor (TigA) involved in the folding of nascent proteins [40] or fusion of MurD with maltose binding protein (data not shown), did not help in solubilizing the recombinant MurD protein. *M. tuberculosis* MurD was finally obtained in its pure active form after cleaving from an *E. coli* expressed fusion with *E. coli* NusA (Table S2), which not only provided good solubilizing properties, but also achieved a very high expression level, thus making it a very good carrier for solubilizing large proteins.

Significant loss in specific activity was observed for all Mur synthetases at temperatures above 40°C, with the enzymes becoming increasingly unstable at higher temperatures. Furthermore, these proteins were highly sensitive to pH, showing optimal activity at alkaline pH 8.0–8.5, as also seen earlier in the cases of MurD [31] and MurE [15], [41], [42]. We further showed that a concentration of UDP sugar higher than 0.2 mM led to significant inhibition of the ligation activity. Similar inhibition was also observed for substrates UDP-MurNAc-L-Ala and UDP-MurNAc-L-Ala-γ-D-Glu-m-DAP at concentrations higher than 0.1 mM in *E. coli* [43]. Therefore, conditions that may result in the increase of intracellular levels of these substrates, may lead to a decrease in the activity of Mur synthetases, thus presumably regulating the rate at which cell wall PG synthesis occurs at different physiological stages or under stress in *M. tuberculosis*. Magnesium or manganese cations were essential for high levels of Mur synthetase activities as assay mixtures which were either devoid of any cation or contained 1–50 mM CaCl_2_, CdCl_2_, CuCl_2_, FeCl_2_, NiCl_2_ and BaCl_2_ showed negligible activity. Indeed K^+^ and NH_4_^+^ gave slightly more activity, however Mg^2+^ is proposed to be central to the activity of Mur synthases [44], and there is no obvious trend from ionic radii or ligand geometry as to which ions are capable of demonstrating activity. Furthermore, the turnover numbers were found to be very similar for all *M. tuberculosis* Mur synthetases under optimal conditions *in vitro*, but lower than those found for *E. coli* Mur synthetases (Table 1).

It was interesting to observe that MurC synthetase from *M. tuberculosis* showed activity not only with L-Ala and Gly as found previously [13], but also with L-Ser as confirmed by HPLC and LC-MS. Activity with L-Ser has been observed for the MurC synthetase of *E. coli* [27], *S. aureus* [45] and *Chlamydia trachomatis* [46]. The comparatively lower specificity for both L-Ser and Gly may be a possible reason for L-Ala being the preferred substrate. Interestingly, PG composition studied in *E. coli* showed neither L-Ser nor Gly in the intracellular pool despite similar *in vitro* specificity [47], suggesting that perhaps only L-Ala is successfully incorporated into PG *in vivo*. Whether a downstream control for composing the correct peptidoglycan precursor exists, where the later Mur synthetases are unable to use UDP-MurNAc-Gly or UDP-MurNAc-L-Ser or derivatives as substrates for ligating their amino acids, remains to be experimentally confirmed for *M. tuberculosis*.

The broad phylogenetic distribution and conservation of gene order and gene content of the *dcw* cluster [48], together with the association of genes involved in cell division and cell wall PG biosynthesis, suggests interactions between genes. To identify the genes co-transcribed with Mur synthetases, we carried out RT-PCR in *M. bovis* BCG, which showed no intergenic amplification between *PE_PGRS38-Rv2161c, Rv2159c-murE* and *ftsQ-ftsZ*. This suggested that *Rv2161c, murE* and *ftsZ* are the first genes on adjacent transcripts, and that the *dcw* operon spans from *murE* to *ftsQ*. Multiple promoters have been identified for *ftsZ* earlier [49], which further support our analysis. The hypothetical protein *Rv2160c*, a putative tetR-family transcriptional regulator found only in pathogenic mycobacteria and positioned on the adjacent transcript, may be involved in the regulation of the genes of the *dcw* cluster.

Phosphorylation by STPKs has recently emerged as a major physiological mechanism for *M. tuberculosis* adaptation to host stimuli [16], [50], [51]. PknA and PknB have been reported to regulate cell division processes and cell wall biosynthesis through phosphorylation of target proteins such as FtsZ [52], Wag31 [53], KasA and KasB [54] and GlmU [55]. *In vitro* studies have also shown that MurC from *Corynebaterium glutamicum* [56] and MurD from *M. tuberculosis* [57] interact with PknA, while PknB is responsible for positioning of the PG biosynthesis protein PBPA in *M. tuberculosis* [58], and that its deletion impacts the UDP-sugar substrate concentrations and hence the cell wall synthesis [59]. Our studies showed that all four Mur synthetases interacted with both PknA and PknB, which makes them likely substrates for their phosphorylation by these STPKs. This suggested that the signal transduction mediated by PknA and PknB are likely to act as a switch for the PG biosynthesis pathway or effect phosphorylation-regulated protein-protein interactions during PG biosynthesis. Though a similar signature phosphorylation motif (X-X-X-X-T-Q-X-hydrophobic-hydrophobic) has been proposed for PknA and PknB [53], these kinases differ in their substrate specificity at the residues adjacent to the phosphoacceptor threonine [60]. This may be a possible explanation towards the variation in the intensity of interaction observed between PknA and PknB with Mur synthetases.

Due to the lack of validation of Mur synthetases with inhibitors, and more recently by interactions observed between MurF, MraY and MurG in *Caulobacter crescentus* [61], there have been speculation of the possible existence of a multi-enzyme complex between the Mur synthetases [62] to explain the lack of activity of Mur inhibitors *in vivo*. Our results however, for the first time indicate that this may not be the case in *M. tuberculosis*, as none of the Mur synthetases showed interaction with each other. Besides PknA/B, other Mur synthetase-interacting partners in the PG biosynthesis pathway were also identified. Specific interactions of MurI, DapF and DdlA were observed only with the protein utilizing their respective reaction product as a substrate, indicating a stringent control of the products and substrates for Mur synthetases and their neighbouring dependants. Furthermore, a recent report has identified NamH as the protein responsible for glycolylation of muramic acid [21] and Lipid II being its proposed substrate [20]. However, it has yet to be ascertained at which step of the pathway this inter-conversion occurs, as well as the biological implications or physiological functions of this structural difference in these PG soluble precursors. In our study we observed that the *namH* homologue (Rv3818) interacted exclusively with MurF, which suggests that it may be acting before Lipid I synthesis, with the glycolyl form possibly introduced into the last soluble, cytoplasmic precursor (UDP-MurNAc-pentapeptide). This hypothesis however, has to be confirmed by determining the activities of Mur synthetases in the presence of the glycolylated UDP-sugar substrates.

As cell division and PG biosynthesis genes share the *dcw* cluster, and that the PG biosynthetic protein PBP3 has been reported earlier to interact with both FtsW and FtsZ forming a trimeric complex in mycobacteria [63], it was reasoned that an interaction between the Mur synthetases and cell division proteins could exist. FtsZ, FtsW and FtsQ constitute the core of bacterial division machinery along with other cell division proteins [64]; Wag31, a homologue of DivIVA, regulates polar cell wall synthesis [65], and the *Bacillus* homologue of Rv2147c, has been reported to play a role in septum development [66]. Our investigation showed that these cell division proteins, which are in close proximity to the Mur synthetases on the *dcw* cluster, interacted with all four of the Mur synthetases. This data reveals that there is interplay between these cell division proteins and the Mur synthetases, which could be a way to tightly control the delicate balance between septation and PG elongation during daughter cell formation.

In this study, we have characterised the *dcw* operon in *M. tuberculosis* and gained further insights into the function and regulatory network of Mur synthetases. The results from our study are a step towards inhibiting this network with new target-specific small-molecule inhibitors, which would be potentially effective drugs against TB.

## Supporting Information

Figure S1 **Optimisation of the physical conditions for Mur synthetases.** Effect of increase in temperature (Ai) and stability of Mur synthetases at room temperature (Aii) over 10 days. Effect of different pH and buffers (B) on the activity of MurC (i), MurD (ii) and MurF (iii). X-axis represents days, different temperatures, or buffers used. Y-axis, in all the cases, represents the amount of P_i_ released in pmol/min.
(TIF)

Figure S2 **Confirmation of formation of UDP-MurNAc-L-Ala, UDP-MurNAc-L-Ser and UDP-MurNAc-Gly by MurC of** ***M. tuberculosis*** **.** Reverse-phase C-18 HPLC chromatograms at 268 nm (i) and negative-mode mass spectrometry (ii) of the product reaction with (A) L-alanine (B) L-serine and (C) glycine. The lines in black represent the chromatogram before addition of the enzyme and the lines in red represent the chromatogram after addition and incubation for 1 hr. The mass spectra were recorded for the major peaks (in the red line) of the products of the reaction with L-Ala, L-Ser and Gly.
(TIF)

Table S1 **Constructs prepared in this study.** * Restriction sites are underlined.
(DOCX)

Table S2 **Details of purified Mur synthetases.**
(DOCX)

Table S3 **Primers for RT-PCR.**
(DOCX)
