# Correction: Characterisation of ATP-Dependent Mur Ligases Involved in the Biogenesis of Cell Wall Peptidoglycan in *Mycobacterium tuberculosis*

**DOI:** 10.1371/journal.pone.0301375

**Published:** 2024-03-26

**Authors:** Tulika Munshi, Antima Gupta, Dimitrios Evangelopoulos, Juan David Guzman, Simon Gibbons, Nicholas H. Keep, Sanjib Bhakta

Errors were made in generating some of the plate images in Fig 5 of this article [1].

**Fig 5:**
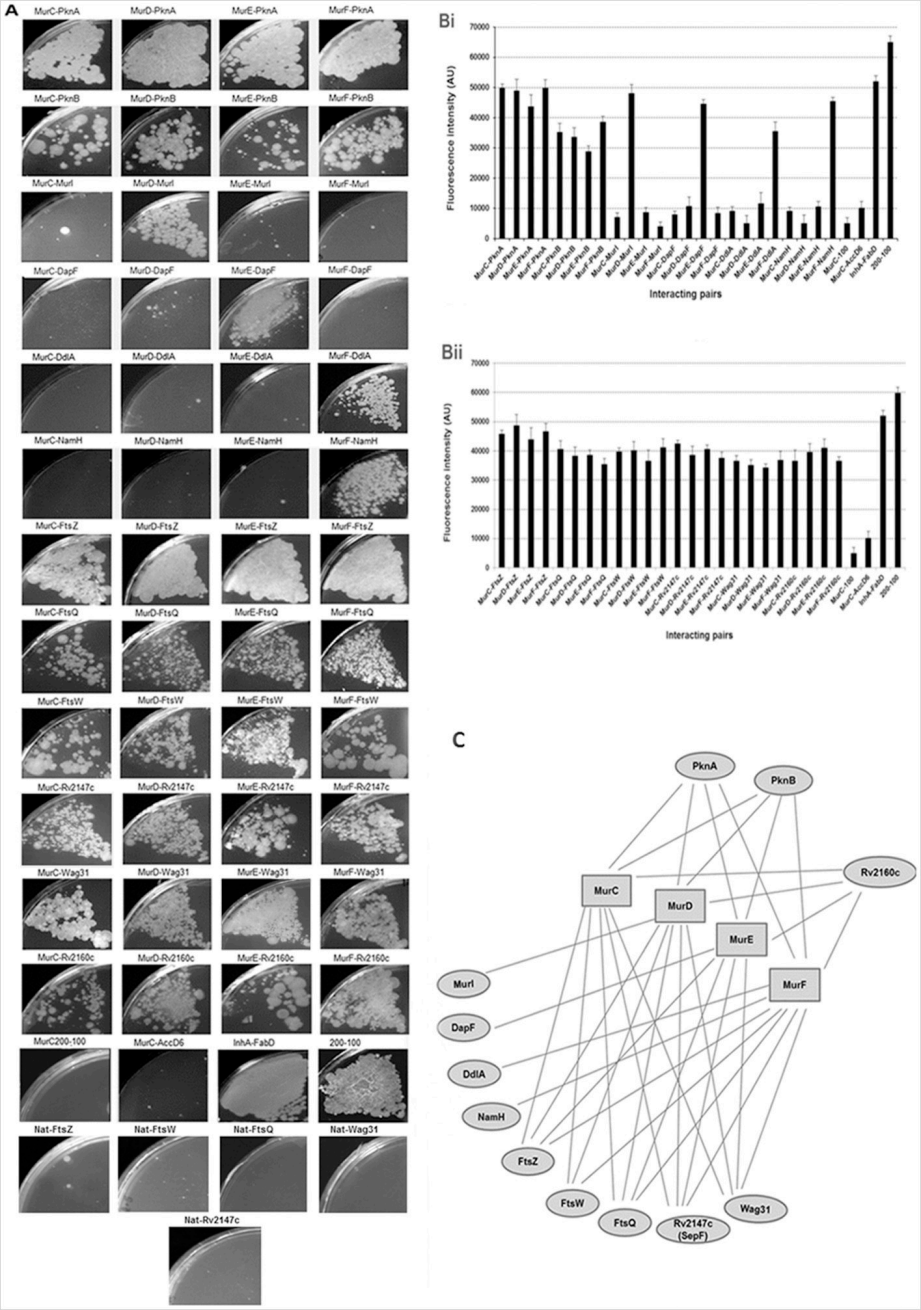
Protein-protein interaction studies of *M*. *tuberculosis* Mur synthetases. (A) Interaction using an M-PFC where growth on TMP plates at 12.5 μg/mL concentration indicated a positive protein-protein interaction, (B) Quantitation of M-PFC interactions by the resazurin assay and (C) representation of final interaction results. Each interaction, by both methods, was assayed in triplicate.

Specifically:

- The MurF-Wag31 panel was erroneously duplicated as the MurE-Wag31 panel in Fig 5A.
- The MurC-Rv2147c panel contains text overlaying the image.

An updated version of Fig 5 is provided with this Correction. The MurE-Wag31 panel has been replaced with correct data from the original experiments, and the MurC-Rv2147c panel has been replaced without the text overlay. The original uncropped images underlying the MurE-Wag31, MurFWag31, and MurC-Rv2147c panels in the updated Fig 5A are available in the S1–S4 Files provided with this notice. The MurF-Rv2147c and MurC-Wag31 panels in Fig 5A are from plates incubated for 8 days to show protein-protein interactions.

The authors did not comment on whether the remainder of the underlying data for this article are still available.

## Supporting information

S1 File Original underlying image of culture plate showing MurE-Wag31 and MurF-Wag31 interaction on day 7. This file includes the original uncropped image for MurC, MurD, MurE and MurF synthetases from *Mycobacterium tuberculosis*, with Wag31 (Day 7).
(TIF)

S2 File Original underlying image of culture plate showing MurE-Wag31 and MurF-Wag31 interaction on day 8. This file includes the original uncropped image for MurC, MurD, MurE and MurF synthetases from *Mycobacterium tuberculosis*, with Wag31 (Day 8—same plate).
(TIF)

S3 File Original underlying image of culture plate showing MurC-Rv2147c interaction on day 7. This file includes the original uncropped image for MurC, MurD, MurE and MurF synthetases from Mycobacterium tuberculosis, with Rv4712c (Day 7).
(TIF)

S4 File Original underlying image of culture plate showing MurC-Rv2147c interaction on day 8. This file includes the original uncropped image for MurC, MurD, MurE and MurF synthetases from *Mycobacterium tuberculosis*, with Rv4712c (Day 8—same plate).
(TIF)
