# Supplementary figures and images for: Characterisation of ATP-Dependent Mur Ligases Involved in the Biogenesis of Cell Wall Peptidoglycan in Mycobacterium tuberculosis
Source: PLoS One. 2013 Mar 21;8(3):e60143. doi: 10.1371/journal.pone.0060143 (PMC3605390; doi:10.1371/journal.pone.0060143)

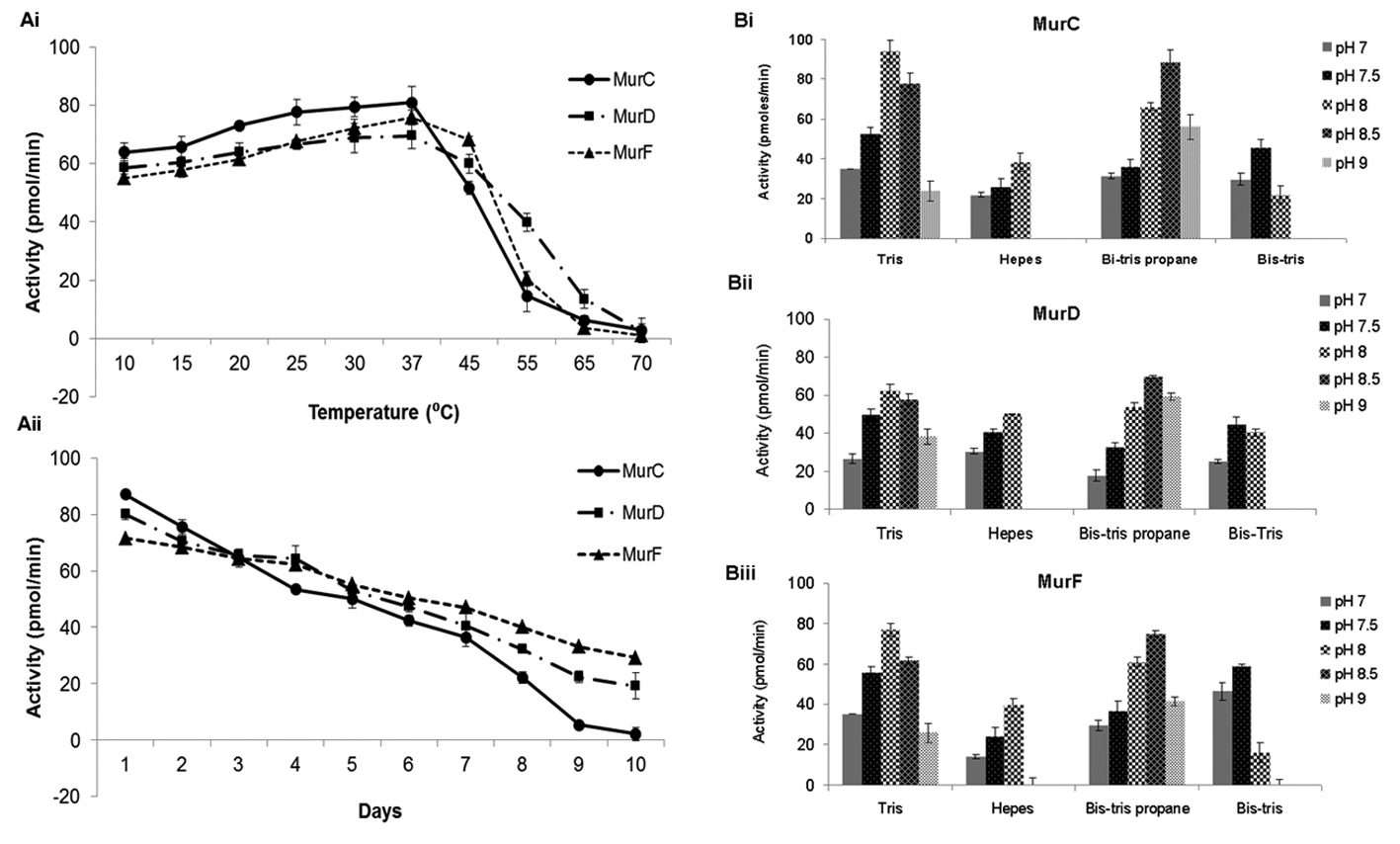

Supplement: Figure S1 — Optimisation of the physical conditions for Mur synthetases. Effect of increase in temperature (Ai) and stability of Mur synthetases at room temperature (Aii) over 10 days. Effect of different pH and buffers (B) on the activity of MurC (i), MurD (ii) and MurF (iii). X-axis represents days, different temperatures, or buffers used. Y-axis, in all the cases, represents the amount of Pi released in pmol/min. (TIF) [file pone.0060143.s001.tif]

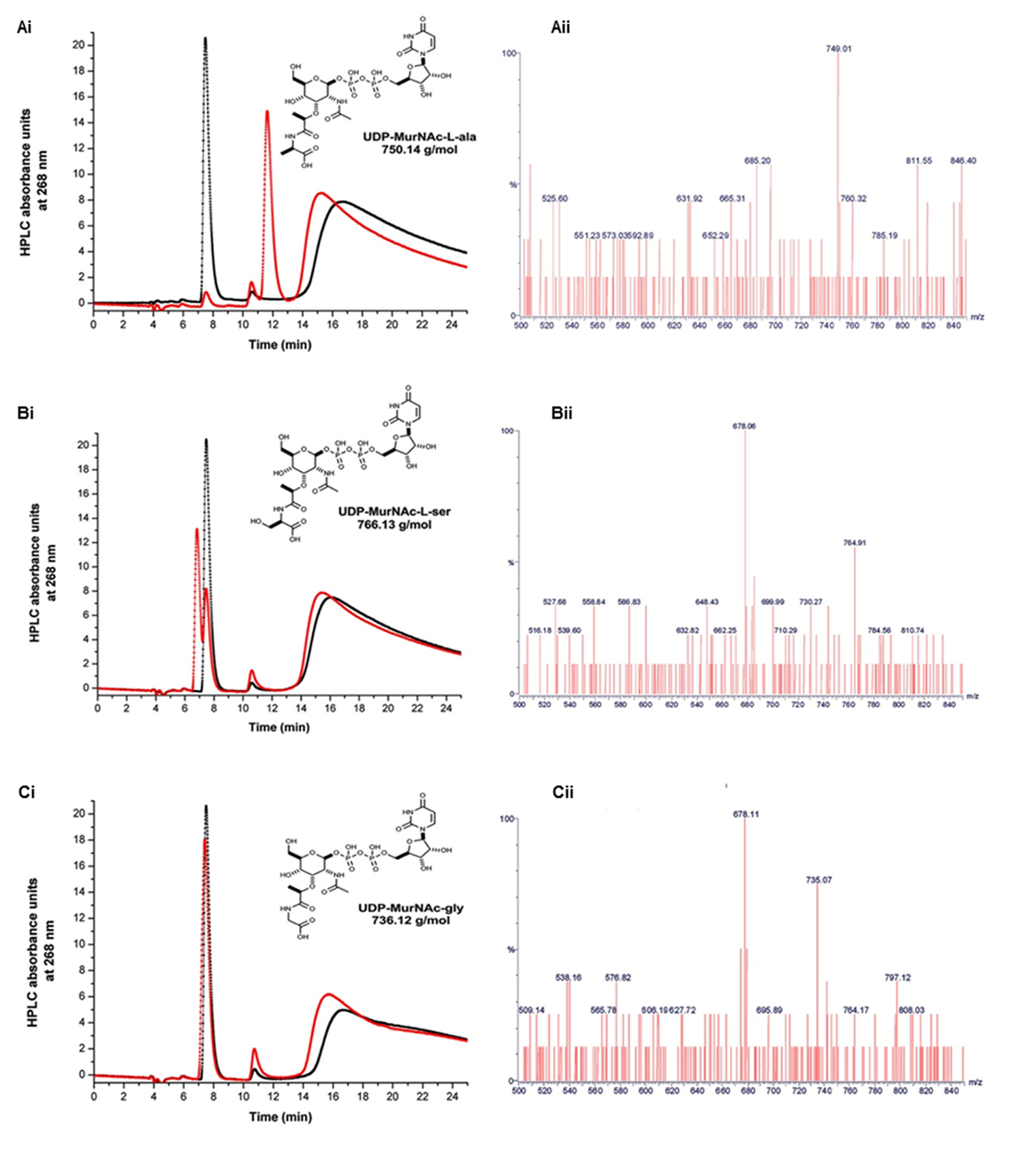

Supplement: Figure S2 — Confirmation of formation of UDP-MurNAc-L-Ala, UDP-MurNAc-L-Ser and UDP-MurNAc-Gly by MurC of M. tuberculosis . Reverse-phase C-18 HPLC chromatograms at 268 nm (i) and negative-mode mass spectrometry (ii) of the product reaction with (A) L-alanine (B) L-serine and (C) glycine. The lines in black represent the chromatogram before addition of the enzyme and the lines in red represent the chromatogram after addition and incubation for 1 hr. The mass spectra were recorded for the major peaks (in the red line) of the products of the reaction with L-Ala, L-Ser and Gly. (TIF) [file pone.0060143.s002.tif]
