# Supplementary material for: Characterisation of ATP-Dependent Mur Ligases Involved in the Biogenesis of Cell Wall Peptidoglycan in Mycobacterium tuberculosis
Source: PLoS One. 2013 Mar 21;8(3):e60143. doi: 10.1371/journal.pone.0060143 (PMC3605390; doi:10.1371/journal.pone.0060143)
Supplement: Table S1 — Constructs prepared in this study. * Restriction sites are underlined. (DOCX) [file pone.0060143.s003.docx]

**Table S1: Constructs prepared in this study**

| **Constructs** | **Description** | **Primers** |
| --- | --- | --- |
| pSBC2 | pET28b+ derivative carrying murC | F-GCCCATATGAGCACCGAGCAGTTGCCG  R-GCCGGATCCTCATCCCAGCACCCCCG |
| p43D | pET43.1b+ derivative carrying murD | F-TTTCGGATTCAGTGCTTGACCCTCTGG  R-TTTAAGCTTCTACCGGATCACCGCGCG |
| pSBC1 | pET28b+ derivative carrying murE | reference 15 |
| pSBC4 | pET28b+ derivative carrying murF | F-CGCCATATGATCGAGCTGACCGTCGC  R-CCCGGATCCTCATGGGCGCACACTCC |
| p31C | pVLT31 derivative carrying His-tagged fusion of murC | sub-cloned from pSBC2 |
| p31F | pVLT31 derivative carrying His-tagged fusion of murF | sub-cloned from pSBC4 |
| pYUBP1 | pYUB76 derivative carrying region P1 | F-TTAAGGATCCTCGCCATGCTGTCCCCG  R-TTAAGGATCCCGGGCCAGCGATGACAC |
| pYUBP2 | pYUB76 derivative carrying region P2 | F-TTTTGGATCCCGGGCTGATCGGCAACG  R-TTTTGGATCCACCGACGCGTGCGTGc |
| murC200 | pUAB200 derivative carrying murC (Bait) | F-GGCAATTGACGTGAGCACCGAGCAG  R-CGTATCGATCCGTCATCCCAGCACC |
| murD200 | pUAB200 derivative carrying murD (Bait) | F-CCTCCAATTGCGCGCATGATCGAGCTG  R-CGCGATCGATGCCTCATGGGCGCACAC |
| murE200 | pUAB200 derivative carrying murE (Bait) | F-CGCCGAATTCGTAGGGTGTCATCGCTG  R-CGGTCAATCGATTCATGCGCGCCGCTC |
| murF200 | pUAB200 derivative carrying murF (Bait) | F-CCTCCAATTGCGCGCATGATCGAGCTG  R-CGCGATCGATGCCTCATGGGCGCACAC |
| pknA100 | pUAB100 derivative carrying murE (Prey) | F-CGCGCGGATCCAATGACCACCCCTTC  R-CGCGCATCGATCTACTGGCCGAACCTC |
| pknB100 | pUAB100 derivative carrying pknA (Prey) | F-GCAGTGGCCACCATGAGCCCCCGAGT  R-GGATCGATTGGTCATTGCGCTATCTC |
| murI100 | pUAB100 derivative carrying murI (Prey) | F-GCCGGATCCTATGAATTCGCCGTTGG  R-GCGCTCATCGATGGCCTAATGAATGC |
| dapF100 | pUAB100 derivative carrying dapF (Prey) | F-CGCGGATCCGGCGCAGATGATCTTC  R-CGCGCATCGATTGCCTGGGATCACC |
| ddlA100 | pUAB100 derivative carrying ddlA (Prey) | F-CGCGGATCCGGTTGTGAGTGCTAAC  R-CACCATCGATCTAGTGCAGGCCCACC |
| namH100 | pUAB100 derivative carrying namH (Prey) | F**-**CGAAAGGATCCTGTGCAGGTCACAAGC  R-CGTAACGTTGCATCATGGCCGTGAAC |
| Rv2160-100 | pUAB100 derivative carrying Rv2160c (Prey) | F-TTGGGGATCCTGTGGGTCGGATACCTG  R-GCCGGATCGATTCATGATTCGACTCCC |
| ftsW100 | pUAB100 derivative carrying ftsW (Prey) | F-TTGGGGATCCTGTGCTAACCCGGTTGCT  R-CCGGATCGATTCACCCGTAACGCTGAC |
| ftsQ100 | pUAB100 derivative carrying ftsQ (Prey) | F-TGGGGGATCCTATGACGGAACACAACG  R-CCGGATCGATCTATTTCACGGTCGGCA |
| ftsZ100 | pUAB100 derivative carrying ftsZ (Prey) | F-TTCCGGATCCTATGACCCCCCCGCACA  R-CCGGATCGATTCAGCGGCGCATGAAG |
| sepF100 | pUAB100 derivative carrying sepF (Prey) | F-GGGGGATCCCGTGAATAGTCACTGTAG  R-GCGGATCGATCTATTGGTAGGCGTAGA |
| wag100 | pUAB100 derivative carrying wag31 (Prey) | F-GGGGGATCCAATGCCGCTTACACCTGC  R-CCCGATCGATCTAGTTTTTGCCCCGGTT |
| nat200 | pUAB200 derivative carrying nat (Bait) | F-GGTACAATTGGCAACATGGCACTGG  R- GCGACAACGTTGAAGCCTTACGG |
| fabD100 | pUAB100 derivative carrying fabD (Prey) | F-GCGGGATCCTCTTAGACACGTGATTG  R-GGCATCGATCCGCGGTTATAGGTTTG |
| inhA200 | pUAB200 derivative carrying inhA (Bait) | F-CGCGCGAATTCACATGACAGGACTGCT  R-GCAATCGATCTAGAGCAATTGGGTGTG |
| accD6100 | pUAB100 derivative carrying accD6 (Prey) | F-GGGATCCGAGACCTGCGATGACAATC  R-GGATCGATCTACAGCGGGATGTTCTTG |
| * Restriction sites are underlined | |  |
