# Supplementary material for: Characterisation of ATP-Dependent Mur Ligases Involved in the Biogenesis of Cell Wall Peptidoglycan in Mycobacterium tuberculosis
Source: PLoS One. 2013 Mar 21;8(3):e60143. doi: 10.1371/journal.pone.0060143 (PMC3605390; doi:10.1371/journal.pone.0060143)
Supplement: Table S2 — Details of purified Mur synthetases. (DOCX) [file pone.0060143.s004.docx]

**Table S2: Details of purified Mur synthetases**

| Proteins | Host | Vectors | Inducer | Antibiotic resistance | Tags | Mass with Histag (kD) | Mass after thrombin cleavage (kD) | Peptide extension up to methionine of gene |
| --- | --- | --- | --- | --- | --- | --- | --- | --- |
| MurC | *P. putida* | pVLT31 | IPTG | Kanamycin | Histag | 53.6 | 51.5 | GSHM |
| MurD | *E. coli* | pET43.1b | IPTG | Tetracycline | NusA & Histag | 110.2 | 51.0 | 1.7kD consisting Enterokinase cleavage site  GSAGSGTIDDDDKSPELVDPM |
| MurE | *P. putida* | pVLT31 | IPTG | Kanamycin | Histag | 57.4 | 55.3 | GSHM |
| MurF | *P. putida* | pVLT31 | IPTG | Kanamycin | Histag | 53.7 | 51.6 | GSHM |
