# Supplementary material for: Characterisation of ATP-Dependent Mur Ligases Involved in the Biogenesis of Cell Wall Peptidoglycan in Mycobacterium tuberculosis
Source: PLoS One. 2013 Mar 21;8(3):e60143. doi: 10.1371/journal.pone.0060143 (PMC3605390; doi:10.1371/journal.pone.0060143)
Supplement: Table S3 — Primers for RT-PCR. (DOCX) [file pone.0060143.s005.docx]

**Table S3: Primers for RT-PCR**

| **Gene overlaps** | **Size amplified (bp)** | **Primers** |
| --- | --- | --- |
| *ftsZ-ftsQ* | 480 | F-AAGAGAAGGCCGAAAAGCTG R-ATGAATTCCACGCCTTTGAG |
| *murC-ftsQ* | 300 | F-GCTACGTCCCGGATTTTTCG R-CCTTCGAATTCTGGGTGCTC |
| *murG-murC* | 345 | F-ATGCGTTGCCGGTAGTCAAT R-GCGTCTGACCCGGAGACCAG |
| *ftsW-murG* | 290 | F-AAACGCAGCCCGCGCGGAAGA R-ACGGCGAGGAACCACAAGAC |
| *murD-ftsW* | 346 | F-AGGTTCCTGTTGCTTGTGTTCT R-ACCCGGGTTAGAGGCTTCTTC |
| *murX-murD* | 319 | F-CTTCCACCACCATTTCGAGT R-ACCGCGTCCGAGGAACTAAC |
| *murF-murX* | 307 | F-GACGCCGCCCTGGCACTACT R-AGAATCGCCACCCCGCCCATC |
| *murE-murF* | 346 | F-GATGCCCAGGTCGTCGAGAT R-CCCGGCAGGGCGAGGAACAG |
| *Rv2159c-murE* | 319 | F-ATCAGGTGACCGACGACGAC R-CCGACCTGATCGGCCAGTGC |
| *Rv2159c-Rv2160c* | 346 | F-ATATCGAAGCGCGCCAACTC R-CTGGCCCACCAGCAGTGTCT |
| *Rv2160c-Rv2161c* | 323 | F-CCCAGGTCTGTGTGTACTCGT R-AACAGGCCCGACTTGCTCAT |
| *Rv2161c-PE_PGRS* | 380 | F-GTTAAAGGCGACGGTGGTAAC R-GTCTTCTCCGGCACATACAGG |
