# Supplementary figures and images for: Correction: Characterisation of ATP-Dependent Mur Ligases Involved in the Biogenesis of Cell Wall Peptidoglycan in Mycobacterium tuberculosis
Source: PLoS One. 2024 Mar 26;19(3):e0301375. doi: 10.1371/journal.pone.0301375 (PMC10965061; doi:10.1371/journal.pone.0301375)

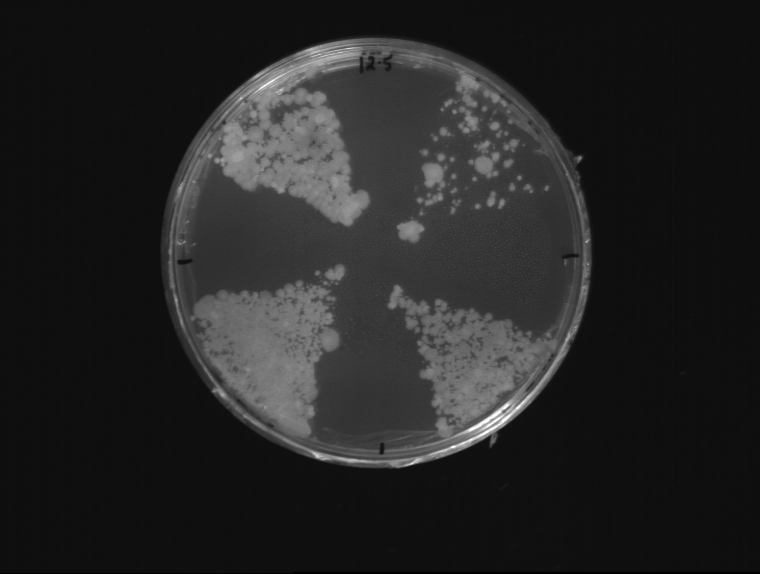

Supplement: S1 File — This file includes the original uncropped image for MurC, MurD, MurE and MurF synthetases from Mycobacterium tuberculosis, with Wag31 (Day 7). (TIF) [file pone.0301375.s001.tif]

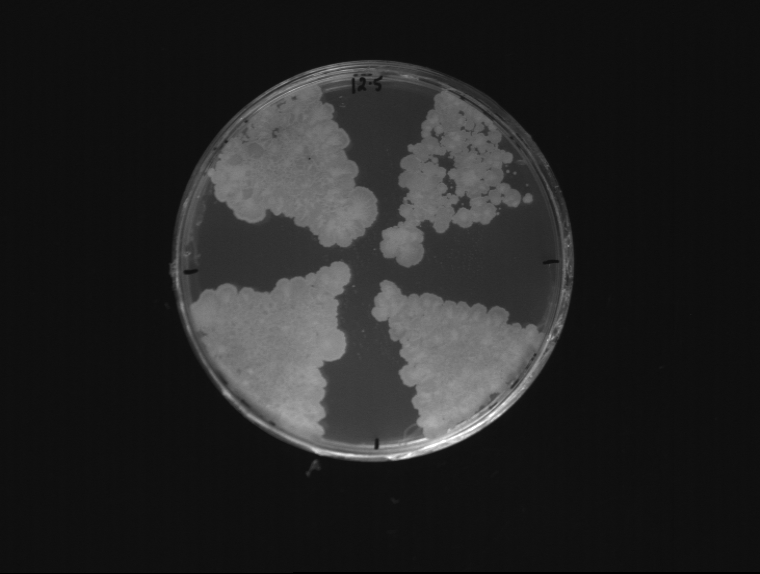

Supplement: S2 File — This file includes the original uncropped image for MurC, MurD, MurE and MurF synthetases from Mycobacterium tuberculosis, with Wag31 (Day 8—same plate). (TIF) [file pone.0301375.s002.tif]

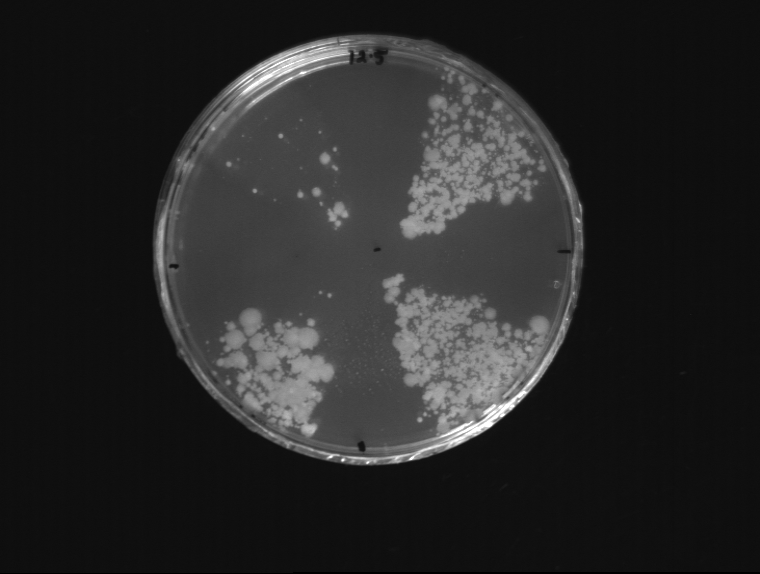

Supplement: S3 File — This file includes the original uncropped image for MurC, MurD, MurE and MurF synthetases from Mycobacterium tuberculosis, with Rv4712c (Day 7). (TIF) [file pone.0301375.s003.tif]

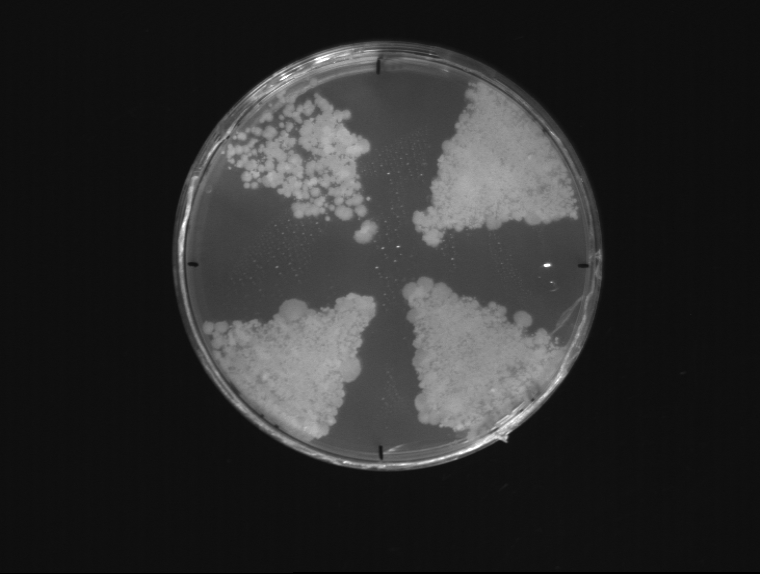

Supplement: S4 File — This file includes the original uncropped image for MurC, MurD, MurE and MurF synthetases from Mycobacterium tuberculosis, with Rv4712c (Day 8—same plate). (TIF) [file pone.0301375.s004.tif]
